# Exploring the Potential of *Myrcia* Genus Essential Oils: A Review of Biological Activities and Recent Advances

**DOI:** 10.3390/molecules29122720

**Published:** 2024-06-07

**Authors:** Eliza de Jesus Barros dos Santos, Fernanda Wariss Figueiredo Bezerra, Luiz Renan Ramos da Silva, Marcilene Paiva da Silva, Oberdan Oliveira Ferreira, Luiza Helena da Silva Martins, Antônio Maia de Jesus Chaves-Neto, Anderson de Santana Botelho, Ravendra Kumar, Pooja Bargali, Karyme do Socorro de Souza Vilhena, Eloisa Helena de Aguiar Andrade, Mozaniel Santana de Oliveira

**Affiliations:** 1 Graduate Program in Biological Sciences, Concentration Area—Tropical Botany, Federal Rural University of the Amazon and Museu Paraense Emílio Goeldi, Av. Perimetral, 1901, Terra Firme, Belém 66077-830, PA, Brazil; elizadejesus098@gmail.com (E.d.J.B.d.S.); luizrenan1@hotmail.com (L.R.R.d.S.); eloisa@museu-goeldi.br (E.H.d.A.A.); 2 Graduate Program of Food Science and Technology (PPGCTA), Institute of Technology (ITEC), Federal University of Pará (UFPA), Belém 66075-110, PA, Brazil; fernandawarissf@gmail.com (F.W.F.B.); luiza.martins@ufra.edu.br (L.H.d.S.M.); 3 Adolpho Ducke Laboratory—Coordination of Botany, Museu Paraense Emílio Goeldi, Av. Perimetral, 1901, Terra Firme, Belém 66077-830, PA, Brazil; arci_paiva@hotmail.com (M.P.d.S.); oberdan@museu-goeldi.br (O.O.F.); andersonbotelho10@hotmail.com (A.d.S.B.); karyme@ufpa.br (K.d.S.d.S.V.); 4 Laboratory of Preparation and Computation of Nanomaterials (LPCN), Federal University of Pará, C. P. 479, Belém 66075-110, PA, Brazil; amchaves@ufpa.br; 5 Department of Chemistry, College of Basic Sciences and Humanities, G.B. Pant University of Agriculture and Technology, Pantnagar 263145, India; ravichemistry.kumar@gmail.com (R.K.); poojabargali8565@gmail.com (P.B.)

**Keywords:** *Myrcia*, natural products, essential oil, volatile compounds, biological applications

## Abstract

The present study provides a comprehensive analysis of the chemical composition of essential oils from species of the *Myrcia* genus and their applications. The compiled results highlight the chemical diversity and biological activities of these oils, emphasizing their potential importance for various therapeutic and industrial applications. The findings reveal that *Myrcia* essential oils present a variety of bioactive compounds, such as monoterpenes and sesquiterpenes, which demonstrate antimicrobial activities against a range of microorganisms, including Gram-positive and Gram-negative bacteria, as well as yeasts. Furthermore, this study highlights the phytotoxic activity of these oils, indicating their potential for weed control. The results also point to the insecticidal potential of *Myrcia* essential oils against a range of pests, showing their viability as an alternative to synthetic pesticides. Additionally, species of the genus *Myrcia* have demonstrated promising hypoglycemic effects, suggesting their potential in diabetes treatment. This comprehensive synthesis represents a significant advancement in understanding *Myrcia* essential oils, highlighting their chemical diversity and wide range of biological activities. However, the need for further research is emphasized to fully explore the therapeutic and industrial potential of these oils, including the identification of new compounds, understanding of their mechanisms of action, and evaluation of safety and efficacy in different contexts.

## 1. Introduction

*Myrcia*, a genus within the Myrtaceae family, offers a wide range of species valued for their medicinal and culinary uses. With leaves and fruits as the primary components, *Myrcia* species are extensively employed in traditional medicine and as flavor enhancers in culinary practices. In Brazil, where over 400 species thrive across diverse regions, *Myrcia* serves as a crucial reservoir of biodiversity, enriching the nation’s natural heritage. A distinctive feature of *Myrcia* species is their abundant essential oil content, primarily composed of mono- and sesquiterpenes, alongside phenolics and others. These bioactive compounds confer a broad spectrum of biological properties to *Myrcia* extracts, including antioxidant, anti-inflammatory, antimicrobial, and antiviral effects. The prevalence of terpenoids underscores the therapeutic potential of *Myrcia* essential oils, offering benefits against oxidative stress, inflammation, and microbial infections [1,2,3].

The extraction technique can be one of the factors that influence the chemical composition of essential oils. Studies on obtaining essential oils from species of the *Myrcia* genus have reported that they are obtained using techniques such as hydrodistillation (HD) and steam distillation (SD). These studies indicate that the HD technique is the one with the higher mass yields when compared to SD. The maximum yield observed for HD extraction of *Myrcia multiflora* was 0.36% (*m*/*m*), while for SD extraction, the yield observed was 0.08% (*m*/*m*) for the same sample [4]; however, the sampling period of the species can be a determining factor that causes variation in the chemical composition of the essential oils. These variations can directly impact their biological activities, as well as their industrial and pharmaceutical applications. Research carried out by Ferreira et al. [5] analyzed the influence on the chemical composition of essential oils obtained from the species *Myrcia eximia* DC, collected in 2017 and 2018; the results obtained by the authors revealed that the time of collection can have a significant influence both qualitatively and quantitatively on the chemical composition of the essential oils of this species.

Among the applications are properties such as antioxidant, antimicrobial, insecticidal, hypoglycemic effects, and phytotoxic. In molecular biology, degenerative processes are associated with the occurrence of excess free radicals, which promote oxidative processes that are harmful to the human body. Plant species are capable of producing compounds such as terpenes, carotenoids, phenols, flavonoids, and anthocyanins, with antioxidant properties capable of neutralizing free radicals, which have a negative impact on the biological cells of living organisms [6]. Some species of the Myrtaceae family reported in the literature have exhibited strong antioxidant properties, which can be utilized in the pharmaceutical and cosmetic industries due to their antioxidant potential [3,7,8].

The genus *Myrcia* is widely acknowledged for its hypoglycemic properties, with numerous species being prominently utilized in traditional medicinal practices for managing diabetes. Research indicates that extracts derived from these plants possess inhibitory effects against the α-glucosidase enzyme, a pivotal player in carbohydrate metabolism. This suggests promising potential in regulating blood glucose levels, which is crucial in diabetes management [9].

Moreover, essential oils (EOs) obtained from species within the Myrtaceae family exhibit a spectrum of activities, including cytotoxicity. The essential oil of *M. floribunda* showed moderate phytotoxicity, in addition, when it came to assessing the toxic effect on the crustacean *Artemia salina*, and the essential oil of *M. floribunda* was classified as moderately toxic to *A. salina* (LC_50_ = 82.96 µg.mL^−1^), while the essential oil of *Myrcia sylvatica* was classified as highly toxic (LC_50_ = 2.74 µg.mL^−1^) [10]. According to Ramos et al. [11], an essential oil is classified as toxic when the LC50 is less than 80 µg.mL^−1^; cytotoxicity is considered moderate when the LC_50_ is between 80 and 250 µg.mL^−1^, and it is considered non-toxic or mildly toxic when the LC_50_ is greater than 250 µg.mL^−1^. The essential oil of *M. floribunda* stood out due to its ability to neutralize the DPPH radical, showing a higher potential than that observed for the Trolox standard (*p* < 0.0001). This result indicated that the essential oil of *M. floribunda* has antioxidant potential, and its activity, according to the author, can be attributed to the chemical composition of the essential oil, which is rich in monoterpenes with antioxidant activity. These findings contribute significantly to our understanding of the genus; however, there remains a considerable need for further exploration to uncover the additional biological properties of *Myrcia* essential oils [10,12,13,14].

This study serves as a comprehensive compilation of the latest insights into the chemical composition and diverse biological activities associated with the essential oils derived from *Myrcia* species. By synthesizing this knowledge, it provides invaluable guidance for future research endeavors aimed at unraveling the full therapeutic potential of *Myrcia* essential oils.

## 2. Methods 

**Figure 1:**
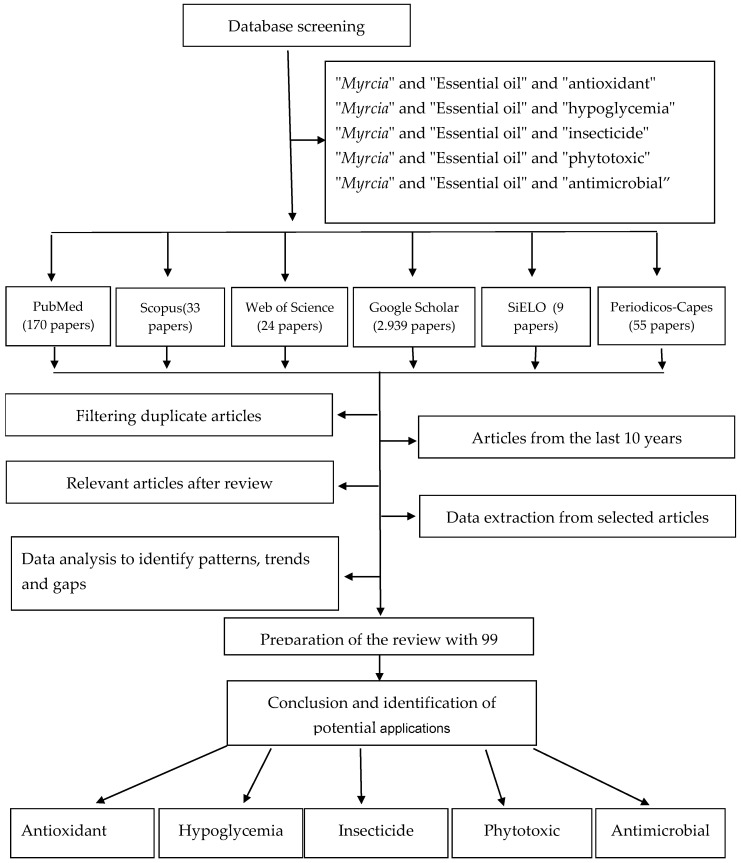
Flow chart of article selection process in the present review.

Figure 1 shows the search and selection methodology for the articles cited in this paper. Different databases were used, and the selection was based on the relevance of the work and recommendations related to the topics covered in this paper.

## 3. Botanical Characteristics

The Myrtaceae family is one of the most important in humid tropics and is widely distributed in South America, Australia, and tropical Asia [2]. Some authors claim that besides the genus *Myrcia*, three other genera of Myrtaceae, namely *Calyptranthes*, *Marlierea*, and *Gomidesia*, are synonyms of this genus [15].

In Brazil, the genus *Myrcia* is represented by over 400 species, found in a variety of environments ranging from dense forests to drier areas. And 304 of these species are exclusive to Brazil, meaning they are not found anywhere else in the world [16]. This highlights not only the richness of *Myrcia* in the country but also the importance of the genus’ diversity to the complexity of Brazilian ecosystems, for example, *Myrcia elevata*, *Myrcia cuprea*, *Myrcia intons*, *Myrcia fasciculata*, *Myrcia neospeciosa*, *Myrcia magna*, *Myrcia uaupensis*, *Myrcia cuspidata*, *Myrcia lepida*, *Myrcia laruotteana Myrcia revolutifolia*, *Myrcia clusiifolia*, *Myrcia saxatilis*, *Myrcia bicarinata*, *Myrcia neosuaveolens*, *Myrcia bicarinata*, *Myrcia* cf. *obversa*, *Myrcia hexasticha*, *Myrcia isaiana*, *Myrcia plusiantha*, *Myrcia tomentosa*, *Myrcia hatschbachii*, and *Myrcia pileata* [17,18,19].

The botanical genus *Myrcia* is monophyletic, meaning it shares a common ancestor and includes a large number of species. Its distribution is comprehensive, extensively occupying the American tropical territory and extending across almost all phytogeographical domains [20]. Diversity is a prominent characteristic of this genus, with approximately 800 species identified in a broad geographic area, from Central America to tropical regions [15,21].

Each species of the genus *Myrcia* displays significant variations in its phenological patterns, influenced by intrinsic factors such as genetic characteristics and extrinsic factors including climatic and microenvironmental conditions. Seasonal responses, such as flowering, fruiting, budding, and leaf fall, often adjust to climatic changes, revealing a temporal synchrony that can be interpreted as an adaptive strategy. Micro environmental conditions, such as cardinal orientation and surrounding vegetation structure, play a crucial role in these phenological patterns [22]. 

The genus *Myrcia* comprises shrubs or small- to medium-sized trees, whose branching follows a dichotomous pattern. Young branches exhibit varied characteristics, such as being flattened, ridged, winged, or pubescent. Their leaves are opposite or simple, with well-defined veins and entire margins, and can be leathery or membranous, without stipules. Trichomes are generally simple, although some species may have bifurcations. Flowers are often arranged in panicles. Before flowering, the calyx of the flowers is closed, opening during flowering, with petals usually absent or small. The reproductive organs have numerous stamens and an ovary with two cells, each containing two ovules. The fruits are berries with 1 to 4 seeds, covered by the basal portion of the calyx, and the seeds tend to be subglobose, with large, thin, and often twisted cotyledons [17,23,24].

*Myrcia* presents its inflorescence in the form of a panicle, which refers to an arrangement of flowers where multiple branches radiate from a central point, presenting a structure resembling a panicle. This structural characteristic facilitates flower distribution, contributing to pollination and, consequently, promoting a high plant reproduction rate. Additionally, the panicle-type inflorescence can exhibit a variety of sizes and shapes, contributing to morphological diversity within the genus [25,26]. 

Species of the genus *Myrcia* exhibit aromatic characteristics due to the production of essential oils as part of their secondary metabolism. These essential oils are composed of volatile substances that impart fragrance to the plants and are stored in specialized structures called secretory cavities. These cavities are found in various parts of the plant, such as leaves, flowers, fruits, stems, or roots, and are responsible for the production, storage, and release of these aromatic substances. Secretory cavities are essential for the adaptation and survival of *Myrcia* species in their natural habitats [27,28,29,30,31]. The genus *Myrcia* is recognized for the presence of several species that produce volatile compounds [32], such as *M. palustres*, *M. guianensis*, *M. sylvatica*, *M. rostrata*, among others [33,34,35,36].

## 4. Chemical Composition of Essential Oils

The essential oils of the *Myrcia* genus exhibit chemovariability, which may be associated with factors such as climatic data, foliar nutrients, aerial parts, habitat, collection time, and also the different types of extraction techniques for these volatile oils [37]. Regarding this diversity in chemical profile, it is possible to observe in Table 1 the major compounds found in the essential oils of some species of the genus, with the presence of monoterpenes, sesquiterpenes, and other chemical classes.

Monoterpenes are natural organic compounds that represent a large group in the field of chemistry [38]. In this study, it was possible to observe that this chemical class characterized the profile of the essential oils of *Myrcia lundiana* (MLUN19), *M. lundiana* (MLUN23), *M. mollis*, *M. ovata*, and *M. Paivae* [7,13,39,40]. 

Sesquiterpenes are classes of compounds found in nature, which are present in both aromatic plants and marine life [41]. In this research, these organic compounds were found to be predominant in the essential oils of various species such as *Myrcia eximia* (Specimen B) [5], *M. hatschbachii* [42], *M. loranthifolia* [43], *M. multiflora* (Specimen A, B and C) [4], *M. oblongata* [44], *M. sylvatica* [10,45], *M. rostrata* [46], *M. splendens* [47,48], *M. vitoriana* [49], and finally, the samples of *M. tomentosa* [3,50]. 

Table 1 presents a comprehensive view of the diversity of plant species of the genus *Myrcia*, collected in various regions, along with the main chemical components extracted and their percentages. Among the species listed, *M. eximia* (Specimen A and B) from Magalhães Barata, Pará, Brazil, stands out, with hexanal (26.09%) and (*E*)-caryophyllene (20.3%) as major compounds, respectively. *M. guianensis*, collected in Rio Verde, Goiás, Brazil, presents methyl salicylate (11.13%) and Geraniol (8.03%) as predominant components. Another notable species is *M. hatschbachii*, from Paraná, Curitiba, Brazil, with trans-calamenene (19.10%) and (*E*)-caryophyllene (10.96%) as main compounds. Furthermore, *M. loranthifolia*, from Igarassu, Pernambuco, Brazil, is marked by (E)-caryophyllene (47.54%) and α-Humulene (9.22%) as major components. This chemical diversity reflects the influence of environmental and geographic factors on the chemical composition of plants, highlighting the importance of an integrated approach to understanding their biology and potential application.

Other species include *M. lundiana*, collected in Itabaiana, Sergipe, Brazil, presenting Isopulegol (40.29%) and Iso isopulegol (15.49%) as the main compounds in the chemotype isopulegol (MLUN19) and Citral (23.43%) and 1,8-Cineole (16.43%) in the citral chemotype (MLUN23). *M. mollis*, from Gonzanamá, Loja Province, Ecuador, has (*Z*)-caryophyllene (16.6–16.8%) and 1,8-Cineole (10.4–11.6%) as highlighted components. *M. multiflora*, collected in Magalhães Barata, Pará, Brazil, presents different specimens, with (*E*)-nerolidol (44.4%) and (*E*)-γ-Bisabolene (10.64%) in Specimen B and (*E*)-nerolidol (92.21%) in Specimen C, among other compounds. This variety of chemical composition highlights the richness and complexity of plants of the *Myrcia* genus, offering vast potential for applications in various areas, from the pharmaceutical industry to cosmetology and aromatherapy.

**Table 1:** Main major compounds of *Myrcia* essential oils. The analyses of the essential oils presented in the table were carried out using Gas Chromatography Coupled with Mass Spectrometry (GC/MS).

Species	Collection Site	Plant Part	Extraction Type	Major Components	References
*Myrcia eximia* (Specimen A)	Magalhães Barata, Pará, Brazil	Leaves	HD	hexanal (26.09%), (*E*)-Caryophyllene (20.3%), and (2E)-hexenal (6.63%).	[5]
*M. eximia* (Specimen B)	Magalhães Barata, Pará, Brazil	Leaves	SD	Caryophyllene oxide (22.16%), α-Copaene (10.98%), and 14-Hydroxy-9-epi-(E)-caryophyllene (7.84%)	[5]
*M. guianensis*	Rio Verde, Goiás, Brazil.	Flores	HD	methyl salicylate (11.13%), geraniol (8.03%), and eugenol (8.12%)	[51]
*M. hatschbachii*	Parana, Curitiba, Brazil	Leaves	HD	trans-calamenene (19.10%), (E)-caryophyllene (10.96%), and spathulenol (5.03%).	[42]
*M. ovata*	Japaratuba, in the state of Sergipe, Brazil	Leaves	HD	nerolic acid (50.56–60.63%), geraniol (73.64–77.07%) neral (18.21–28.39%), geranial (36.96–48.48%), and (*E*)-nerolidol (26.97–58.27%)	[43]
*M. lundiana* chemotypes isopulegol (MLUN19)	Itabaiana, Sergipe, Brazil.	Freshly leaves	HD	Isopulegol (40.29%), Iso isopulegol (15.49%), and 1,8-cineol (14.16%)	[13]
*M. lundiana* chemotype citral (MLUN23)	Itabaiana, Sergipe, Brazil.	Freshly leaves	HD	Citral (23.43%), 1,8-cineol (16.43%), and Nerolic acid (16.42%)	[13]
*M. mollis*,	Gonzanamá, province of Loja, Equador	Leaves	SD	limonene (5.3–5.2%), 1,8-cineole (10.4–11.6%), and (Z)-caryophyllene (16.6–16.8%)	[39]
*M. multiflora* (Specimen A)	Magalhães Barata, Pará, Brazil	Leaves	HD	α-bulnesene (26.79%), pogostol (21.27%), and δ-amorphene (6.76%)	[4]
*M. multiflora* (Specimen B)	Magalhães Barata, Pará, Brazil	Leaves	HD	(E)-nerolidol (44.4%), (E)-γ-bisabolene (10.64%), and (E,E)-α-farnesene (8.19%)	[4]
*M. multiflora* (Specimen C)	Magalhães Barata, Pará, Brazil	Leaves	HD	(E)-nerolidol (92.21%), (E,E)-α-farnesene (3.28%), and E-caryophyllene (1.11%)	[4]
*M. oblongata*	Cascavel, Paraná, Brazil.	Leaves	HD	caryophyllene oxide (22.03%), and trans-verbenol (11.94%).	[44]
*M. ovata*	Japaratuba, Sergipe, Brazil	Leaves	HD	Geranial (40.10%), Neral (28.39%), and Citronellal (9.19%)	[40]
	Guaramiranga, Ceará, Brazil	Leaves	HD	Geranial (52.60%) and neral (37.14%)	[52]
*M. paivae*	Peixe-Boi, Pará, Brazil	Leaves	HD	terpinolene (14.70%), α-phellandrene (14.69%), and γ-terpinene (9.64%)	[7]
*M. sylvatica*	Santarém, Pará, Brazil	Leaves	HD	β-selinene (9.96%), cadalene (9.36%), α-calacorene (9.17%)	[45]
	Bujaru, Pará, Brazil	Leaves	HD	(Z)-α-trans-bergamotene(24.57%), α-sinensal (13.44%), and (Z)-α-bisabolene (8.33%)	[10]
*M. rostrata*	Alagoinhas, Bahia, Brazil	Leaves	HD	carotol (17.68%), germacreno B (7.28%), and (*E*)-caryophyllene (6.45%)	[46]
*M. splendens*	Manaus, Amazonas, Brazil	Leaves	HD	trans-nerolidol (67.81%), α-bisabolol (17.51%), and β-caryophyllene (4.21%	[48]
	Sergipe, Brazil	Fresh leaves	HD	bicyclogermacrene (15.4%), germacrene D (8.9%), and E-caryophyllene (10.1%)	[47]
*M. vittoriana*	community in southeastern Brazil	Fresh leaves	HD	germacrene D (21.90%), germacrene B (17.30%), and bicyclogermacrene (11.90%)	[49]
*M. tomentosa* (specimen A)	Magalhães Barata, Pará, Brazil	Leaves	HD	γ-elemene (12.52%), germacrene D (11.45%), and (E)-caryophyllene (10.22%)	[3]
*M. tomentosa* (specimen B)	Magalhães Barata, Pará, Brazil	Leaves	HD	spathulenol (40.70%), α-zingiberene (9.58%), and γ-elemene (6.89%).	[3]
*M. tomentosa*	Belo Horizonte, Minas Gerais	Fresh leaves	SD	germacrene D (17.62%), (E)-caryophyllene (15.05%), and δ-cadinene (6.42%)	[50]

HD: Hydrodistillation; SD: Steam distillation.

Other chemical classes were also observed, such as aldehydes characterizing the essential oils of *Myrcia eximia* (Specimen A) [5] and phenylpropanoids in the oils of *M. guianensis* [51]. In summary, this research highlights the relevance of the volatile oils of this genus and their main implications in future studies, particularly in biological applications such as antimicrobial [53], antileishmanial [52], and insecticidal [54].

## 5. Antioxidant Activity

Antioxidant activity refers to the ability of certain substances to inhibit the damaging effects of free radicals and oxidative stress in biological systems. Free radicals are highly reactive molecules that can cause damage to cells, proteins, DNA, and other biomolecules, leading to various diseases and aging processes. This activity helps protect cells and tissues from oxidative damage and is associated with various health benefits, including reducing the risk of chronic diseases such as cardiovascular disease, cancer, and neurodegenerative disorders. 

The antioxidant potential of the investigated substances was assessed by comparing them to Trolox, a water-soluble synthetic analog of vitamin E. This evaluation was based on their ability to inhibit the radical cation ABTS^+•^, which is formed by the reaction between 2,2′-azino-bis (3-ethylbenzothiazoline-6-sulfonic acid) diammonium salt and potassium persulfate. The antioxidants reduce the ABTS^+•^ to ABTS, and the extent of this reduction depends on factors such as antioxidant capacity, concentration, and reaction time. Both TEAC and DPPH assays were employed to evaluate the antioxidant capacities of the essential oils. The DPPH method assesses the ability of EOs to neutralize 1,1-diphenyl-2-picrylhydrazyl. The EO of *M. tomentosa* exhibited significant inhibition of both ABTS^+•^ and DPPH^•^ radicals by 53.6% and 213%, respectively. This study represents the first report on the antioxidant activity of EOs extracted from *Myrcia tomentosa* reported by Franco et al. [3].

De Moraes et al. [7] reported the antioxidant activity of *Myrcia floribunda* and *Myrcia sylvatica* essential oil via ABTS^•+^ and DPPH^•^ methods. The results of their study indicated that the essential oil (EO) of *M. floribunda* exhibited an ABTS^•+^ inhibition rate of 53.27 ± 8.27%, comparable to Trolox (45.74 ± 4.16%) in terms of antioxidant potential. In the DPPH assay, the EO showed an AIR of 81.91 ± 3.46%, surpassing Trolox (50.53 ± 0.52%) significantly (*p* < 0.0001), suggesting excellent antioxidant activity attributed to its high levels of antioxidant monoterpenes. Conversely, *M. sylvatica* EO had a lower ABTS^•+^ AIR of 7.20 ± 0.72% compared to *M. floribunda* and Trolox. However, in the DPPH assay, *M. sylvatica* EO showed comparable AIR (80.55 ± 2.00%) to *M. floribunda* and superior to Trolox (*p* < 0.0001), possibly due to the presence of reactive oxygenated sesquiterpenes. Table 2 summarizes the antioxidant efficacy of various *Myrcia* species using different methods.

## 6. Biological Activities 

### 6.1. Antimicrobial (Fungi and Bacteria)

Essential oils are associated with various biological activities, as seen in species of *Myrcia* (Myrtaceae family), which have been reported to possess antioxidant, anticancer, antiparasitic, anti-inflammatory, antiproliferative, cytotoxic, insecticidal, anticholinesterase, sedative, anesthetic, and antimicrobial properties [40,58,59]. It is important to highlight that these activities may be related to genetic or environmental (edaphoclimatic) variations, such as ambient temperature, light incidence, rainfall, and soil characteristics, which influence the plant’s metabolism and, consequently, the concentration of its secondary metabolites [59].

Essential oils from various *Myrcia* species have been investigated for their use against pathogenic microorganisms affecting humans, animals, and plants. Nine essential oils from *M. ovata* Cambessedes were studied and showed inhibitory and bactericidal effects against *Staphylococcus aureus*, *Bacillus cereus*, *Bacillus subtilis*, *Enterococcus faecalis*, and *Pseudomonas aeruginosa* at concentrations ranging from 3.13 to 25 μL/mL. Gram-positive bacteria were more susceptible to the essential oil than Gram-negative ones. The study emphasized the inhibition and elimination effects of most essential oils against *P. aeruginosa*, a bacterium known for its high resistance to other essential oils and even synthetic antibiotics [40]. 

The antibacterial activity of the essential oil from *M. hatschbachii* D. Legrand leaves was studied using the Minimum Inhibitory Concentration (MIC) test. The essential oil showed moderate activity against *E. faecalis* (500 µg/mL) and weak activity against *S. aureus* (1000 µg/mL) [42]. 

Three specimens of *Myrcia multiflora* (A, B, C) had their essential oils evaluated for antifungal activity against *Candida albicans*, *C. tropicalis*, *C. famata*, *C. krusei*, and *C. auris*. In other studies, it was found that specimen B showed greater sensitivity against the studied yeasts, with inhibition halos ranging from 9 to 11 mm. Specimen A showed an MIC of 0.78 µL/mL against *C. famata* growth and a minimal fungicidal concentration (MFC) of 12.5 against *C. auris*, while specimen C showed a minimal inhibitory concentration of 5 µL/mL against *C. auris* growth and an MFC greater than or equal to 50 against the studied yeasts [4].

The essential oil of *M. oblongata* was studied for its MIC and Minimum Bactericidal Concentration (MBC) against ten *Salmonella* spp. serotypes (*S. Albany*, *S. braenderup*, *S. gafsa*, *S. heidelberg*, *S. idikan*, *S. lexington*, *S. livingstone*, *S. montevideo*, *S. saintpaul*, and *S. senftenberg*). In the study, Santana et al. [55] showed that the essential oil exhibited an MIC and MBC ranging from 437.5 to 7000 µg/mL for the ten serotypes, with better activities against *S. Lexington* (MIC = 437.5 and MBC = 3500) and *S. Saintpaul* (MIC = 875 and MBC = 1750).

The essential oil of *M. ovata* was first evaluated in 2010 for its antimicrobial activity against *Enterococcus faecalis*, *Escherichia coli*, *Helicobacter pylori*, *Pseudomonas aeruginosa*, *Salmonella choleraesuis*, *Staphylococcus aureus*, *Streptococcus pneumoniae*, and *Candida parapsilosis*, as well as biofilm formation by *Enterococcus faecalis*. The authors reported that the essential oil exhibited antimicrobial activity against all tested microorganisms (diameter of inhibition zone = 16 to 36 mm), except for *Helicobacter pylori* (0 mm), and was effective against biofilm formation compared to the control (sterile saline) [60].

The antibacterial activity of the essential oil of *M. splendens* was evaluated against Gram-positive and Gram-negative bacteria, with the MIC determined by the serial microdilution method. The oil showed more satisfactory effects against phytopathogen strains, with greater sensitivity and, consequently, lower MIC value (125 μg/mL) for the Gram-positive *C. michiganensis* subsp. *Nebraskensis*; all Gram-negative bacteria showed resistance to the essential oil, except for *P. syringae* pv. *seringas*, which showed an MIC value of 250 μg/mL. These effects were attributed to the major compounds, the sesquiterpenes trans-nerolidol (67.81%) and α-bisabolol (17.51%) [48].

The antimicrobial activity of essential oils from *Myrcia* species, as well as from other essential oils, is mainly related to the presence of their volatile compounds such as terpenes (mono-, sesqui-, and diterpenes), alcohols, acids, esters, epoxides, aldehydes, ketones, amines, and sulfides [3,5,61].

The antifungal activity is attributed to the lipophilic nature and low molecular weight of these compounds, which can lead to the inhibition of sporulation, germination, and cell death [4,62]. Meanwhile, the antibacterial activity is associated with the ability of essential oils to penetrate the lipid membrane of bacteria, leading to leakage of intracellular components, inhibiting their functional properties and, eventually, leading to cell death [40,61].

It is important to highlight that the antifungal and antibacterial activities of essential oils are also attributed to the synergistic action of minor concentration compounds with the major compounds. Table 3 presents the antifungal and antibacterial activities of essential oils from *Myrcia* species, along with the main chemical compounds found in these oils.

### 6.2. Phytotoxic Activity of Myrcia Genus

Allelochemicals, compounds produced by plants and other species, induce allelopathic (phytotoxic) activity by affecting the growth and development of nearby plants. Allelochemicals can intervene in physiological processes in different ways and exhibit diversified mechanisms of action. Some substances may inhibit seed germination by interfering with water absorption or cell division. Other compounds affect root growth, so plants are unable to absorb nutrients from the soil, or photosynthesis, reducing energy production. Allelochemicals can also affect the metabolism of the host plant, hindering the production of essential enzymes or hormones. Studies also indicate that some of these compounds limit the production of reactive oxygen species, creating conditions of oxidative stress. The effectiveness of allelochemicals varies based on environmental conditions or the concentrations in which they are found [63,64].

Phytotoxic activity refers to the ability of certain substances, such as plant extracts, essential oils, etc. to inhibit the growth of other plants. This activity is often studied in the context of weed control or understanding the interactions between different plant species in ecosystems. Phytotoxic compounds can affect various processes in plants, including germination, root growth, shoot development, and overall plant health. Studying phytotoxic activity helps in identifying natural or synthetic compounds that could potentially be used for weed management or understanding ecological relationships between plants. Vasconcelos et al. [49] reported a macroscopic analysis that involved assessing the germination of *Lactuca sativa* and *Allium cepa*, followed by evaluating germination percentage and root growth. Notably, the essential oil of *Myrcia vittoriana* decreased the germination rate of *A. cepa*. All essential oil concentrations except 375 µg.mL^−1^ reduced the germination speed index of *A. cepa*, with the highest concentration showing the most substantial reduction. The essential oil strongly inhibited aerial growth in *L. sativa* across all concentrations compared to water, with a dose–response relationship evident. At the highest concentration, it caused a 54.8% inhibition in aerial growth, accompanied by yellowish seedling coloration and leaf necrosis. The growth of the aerial part of *Lactuca sativa* was strongly inhibited by the essential oil of *M. vittoriana*.

The phytotoxic activity was assessed by examining the impact of *Myrcia hatschbachii* essential oil on the germination and growth of *Lactuca sativa* seeds. Results revealed that the highest concentration (1%) of the EO exhibited the most pronounced effects on both hypocotyl and radicle growth. All concentrations of the EO hindered hypocotyl growth in *L. sativa*. For the radicle, the EO showed significant growth inhibition at the 1% concentration, while concentrations of 0.001% and 0.01% also displayed adverse effects compared to the control groups (water and 1% polysorbate in water), as presented by Gatto et al. [42]. The study investigated the allelopathic effects of extracts, fractions, essential oils, and isolated chemical compounds (gallic acid and protocatechuic acid) on the germination, radicle, and hypocotyl growth of weed species *Mimosa pudica* and *Senna obtusifolia*. Extracts and fractions were examined at a concentration of 1%, while essential oils and isolated chemical compounds were tested at 15, 30, 45, and 60 ppm. Interestingly, the essential oil inhibited the germination of *M. pudica* [65]. 

### 6.3. Insecticide

Several plants possess defense mechanisms against insects that can harm their growth and development [66]. Among them is the production of chemical compounds with insecticidal properties that are capable of repelling, incapacitating, or even killing insects [67,68]. These compounds can be found in various parts of the plant, such as leaves, flowers, stems, and roots, and play a crucial role in protecting plants against herbivorous insects, helping them survive and successfully reproduce in their natural environment [69,70,71,72].

Many of these natural insecticidal compounds, in addition to protecting the plant itself, can also be used for pest control in agriculture, gardening, and forestry management and can be applied directly to plants as natural extracts, essential oils, or plant-based products [73,74]. Thus, studies of plant insecticidal compounds have been developed to obtain safer and more sustainable pesticides, reducing dependence on environmentally harmful synthetic chemicals and human health risks.

Among these studies are some conducted with the genus *Myrcia*, which demonstrate the insecticidal potential of the species. In a study conducted with extracts and essential oil from the leaves of *Myrcia oblongata* DC, for example, the insecticidal effect on the beetle *Alphitobius diaperinus* was evaluated. In the study, the hexane extract, composed of steroids, triterpenoids, alkaloids, tannins, and flavonoids, and the essential oil showed the highest mortality rates (<95%) against *A. diaperinus* larvae, while the other extracts showed mortality rates above 50%, and the aqueous extract showed no activity. The highest lethality against adult *A. diaperinus* was observed for the essential oil (92.5%), while the extracts had mortality values below 20% [55].

In another study conducted with the methanolic extract of *Myrcia* splendens leaves, insecticidal action against the *Ceratitis capitata* fly species was observed. The activity was observed in extract fractions obtained by HPLC, which had their compounds isolated and identified as myricetin-3-*O*-(6″-*O*-galloyl)-β-galactopyranoside, myricitrin, and quercitrin. Among these, the compound myricetin-3-*O*-(6″-*O*-galloyl)-β galactopyranoside showed moderate activity at 2500 ppm [75].

In another study on the insecticidal potential of the *Myrcia* genus, the insecticidal action of the essential oil from the leaves of two chemotypes of *Myrcia lundiana* and their major compounds, isopulegol and citral, against the *Acromyrmex balzani* ant species was evaluated. Other essential oils from *M. lundiana* showed activity. The oil from the isopulegol chemotype presented LC_50_ values of 2.29 μL/L (CI 2.08–2.49) and LC_95_ of 6.28 μL/L (CI 5.32–7.97), and its major compound, isopulegol (40.29%), presented LC_50_ and LC_95_ values equal to 0.16 μL/L (CI 0.14–0.19) and 1.08 μL/L (0.81–1.58), respectively, while the citral chemotype presented LC_50_ values of 2.35 μL/L (CI 2.13–2.61) and LC_95_ of 9.14 μL/L (CI 7.25–12.61), and its major compound, citral (23.43%), presented LC_50_ and LC_95_ values equal to 0.59 μL/L (CI 0.48–0.69) and 3.83 μL/L (2.87–5.77), respectively [13].

Another species from the genus that had its insecticidal potential evaluated was *Myrcia dictyophylla*. A study conducted with the species’ essential oil against *Aedes aegypti* observed insecticidal action against the arbovirus vector, where an LC_50_ value of 17.6 µg/mL for mosquito larvae after 24 h of exposure and 92.5% knockdown was observed in adult mosquitoes after 4 h of exposure, in addition to repellent action. The studied oil presented three major sesquiterpenes: α-amorphene (19.8%), δ-cadinene (12.5%), and germacrene-D (10.3%) [76]. 

Although incipient, these studies demonstrate the insecticidal potential of the genus *Myrcia*, especially its essential oils, not only in pest control for agriculture but also in controlling disease vector insects. These studies reveal the genus as a promising sustainable strategy in insect control and as a natural alternative to synthetic compounds in the biological control of parasites, highlighting the need for further studies to explore and better elucidate this potential.

### 6.4. Hypoglycemic Potential of Myrcia Species

Diabetes refers to a group of diseases characterized by high blood glucose levels. Generally, it results from a deficiency in the production or action of insulin, resulting in metabolic disorders. Type 2 diabetes mellitus (DM) is the most common form of diabetes, and its main characteristics are insulin resistance, chronic hyperglycemia, and inefficient insulin secretion and action. According to the World Health Organization (2017), diabetes is responsible for the deaths of around 1.6 million people worldwide [77].

The main drugs used to treat DM2 include insulin sensitizers, glucosidase inhibitors, incretin-based therapies, and sodium-glucose co-transporter 2 inhibitors. Some species of plants, fruits, and teas, for example, may emerge as an alternative to traditional pharmacological therapy. So-called dietary supplements can be recommended to prevent and also improve disorders caused by DM2. The treatment of DM2, in an advanced phase, with synthetic drugs and compounds of natural origin cause synergistic interactions resulting in a more efficient treatment [78].

Therefore, knowledge about the properties, chemical composition, and beneficial characteristics of *Myrcia* species, popularly used to treat diabetes, can help offer a complementary natural alternative to the use of synthetic drugs [79].

Among the species of *Myrcia*, plants known as “pedra-hume-caá”, “pedra-ume-caá”, or “insulin plant” are noteworthy. They are known by these designations due to their use by indigenous communities in Brazil to lower blood sugar levels and are commonly used in the treatment of diabetes. In the literature, several species are related to the term “pedra-hume-caá”, including: *Myrcia guianensis* (Aubl.) DC., *Myrcia punicifolia* (Kunth) DC., *Myrcia citrifolia* (Aubl.) Urb., *Myrcia uniflora* DC., *Myrcia multiflora* (Lam.) DC., *Myrcia salicifolia* DC., *Myrcia speciosa* (Amsh.) Mc Vaugh, *Myrcia rubella* Cambess., *Myrcia virgata* Cambess., *Myrcia paivae* O. Berg., *Myrcia laruotteana* Camb., and *Myrcia sylvatica* (G. Mey) DC [37,80,81,82].

A study has shown that extracts obtained from the leaves of *M. guianensis* exhibit inhibition against α-glucosidase, which is the enzyme responsible for the metabolism of carbohydrates and a known target for type 2 diabetes mellitus (DM2), alongside demonstrating nephroprotective and hepatoprotective action. The ethyl acetate extract of *M. guianensis* showed the greatest potential for inhibiting both alpha-glucosidase and PTP1B (protein tyrosine phosphatase). The extract showed IC_50_ values for alpha-glucosidase of 7.80 µg/mL, while for the enzyme PTP1B, the value observed was 3.26 µg/mL [81]. This same chemical extract has been shown to be active against the protein tyrosine phosphatase (PTP1B), which is a recent target for DM2 and is involved in insulin signaling [9]. 

Another species of the genus, also known as the insulin plant, is *M. multiflora* (Lam.) DC., a shrubby species, also known as “pedra-ume-caá”, typical of the Brazilian Amazon region, occurring in the states of Amazonas, Pará, and Amapá [9]. This species is also found in regions of the Brazilian Cerrado, characteristic of mountainous terrain, poor soils, and margins of sandy or non-floodable fields [4]. *M. multiflora* produces a globose fruit, with succulent and edible pulp, highly appreciated by children [83]. The fruits range in color from dark red to purple when ripe, containing one, two, or three seeds, with a bitter taste [84].

Studies involving ripe fruits of *M. multiflora* have shown that they have a high antioxidant potential, supporting the idea that their consumption may result in health benefits for humans. Samples obtained from the fruits were analyzed for non-enzymatic antioxidant content and showed significant amounts of vitamin C, alongside being rich in anthocyanins, flavonoids, and polyphenols [4,84]. Another study has indicated the presence of a substance beneficial for preventing obesity, capable of reducing intestinal lipid absorption [83]. 

Research involving the use of substances known as myrciacitrins obtained from the leaves of *M. multiflora* has shown, in rats, activity against the enzyme aldose reductase, which is responsible for catalyzing and reducing glucose. The dry extract obtained from the infusion of *M. multiflora* leaves showed a glycemic effect in animals induced for 28 days. In the group of animals whose glucose levels were measured at the beginning of and during treatment with 50 mg/kg (body weight of dry extract), there was a 74.5% reduction in glucose levels when compared to the results of the levels before the start of treatment. At the end of the treatment, after 28 days, the author reported that there was an 11% reduction in glucose levels compared to the beginning of the tests. According to him, this result indicated that the use of the dry extract of *M. multiflora* is capable of maintaining the hypoglycemic effect [85,86].

**Figure 2:**
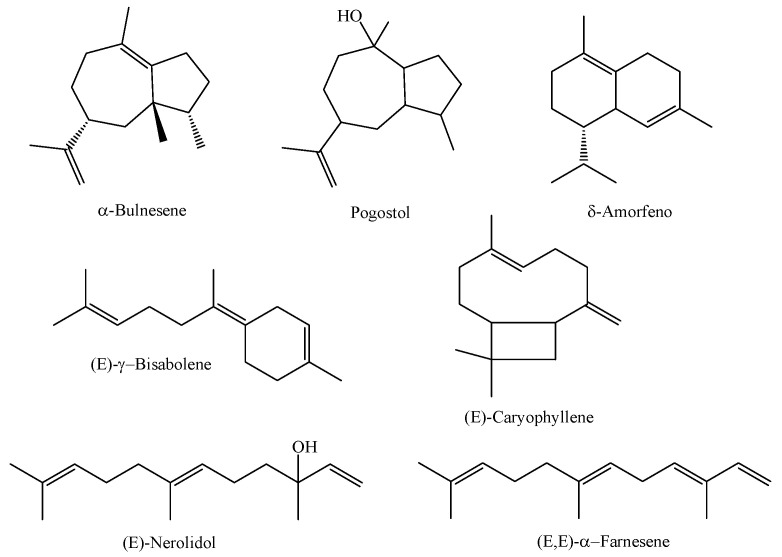
Major constituents of essential oils from *M. multiflora* species collected in the state of Pará.

In a study involving the essential oil obtained from the leaves of three species of *M. multiflora* (referred to as A, B, and C) collected in different areas of a secondary forest in the coastal region of the state of Pará in the city of Magalhães Barata, the essential oils obtained from the species referred to as A (collected in 2017 in an area of the secondary forest -capoeira-), B (collected in 2017 in areas of swidden planting exposed to sunlight), and C (collected in 2019 in an area of the secondary forest -capoeira-) were characterized as having α-bulnesene (26.79%), pogostol (21.27%), and δ-amorphene (6.76%) as major compounds for chemical type A, (*E*)-nerolidol (44.4%), (*E*)-γ-bisabolene (10.64%), and (*E*,*E*)-α-farnesene (8.19%) for chemical type B, and the substances (*E*)-nerolidol (92.21%), (*E*,*E*)-α-farnesene (3.28%), and (*E*)-caryophyllene (1.11%) were the major constituents identified in the essential oil of *M. multiflora*—chemical type C. The research also reported a significant difference between the chemical profiles of the *M. multiflora* species studied [4]. The chemical structures of the main constituents of *M. multiflora* (A, B, and C) are shown in Figure 2.

The essential oils of *M. multiflora* are rich in (*E*)-nerolidol, which is the main component of the essential oil of two chemotypes (B and C) collected in the state of Pará [41]. A study showed that nerolidol significantly improved insulin levels and body weight when administered to obese and diabetic animals at a dose of 25 mg/kg (body weight/day) for 28 days. As a result, high glucose and glycated hemoglobin levels were reduced. In addition, the study reported that during the use of nerolidol, liver integrity was preserved, and the regulation of insulin-dependent glucose transport was improved in skeletal muscles due to the activation of GLU4 (Glucose transporter type 4) [87,88]. It is also worth mentioning that another substance with reported antihyperglycemic activity in the literature is caryophyllene, also identified in the essential oil of *M. multiflora*, which showed significant antidiabetic effects on glucose homeostasis in diabetic rats at a dose of 200 mg/kg (body weight) [89].

*M. bella Cambess.*, also popularly known as “mercurinho”, is a common species in savanna fragments and easily found in the Brazilian Cerrado [86]. This species is widely used in traditional medicine due to its use as a phytotherapeutic medication for the treatment of gastrointestinal disorders and diabetes mellitus [90]. The species is a source of phenolic compounds and flavonoids, with fractions rich in flavonoids showing cytotoxic effects against osteosarcoma cells [91]. 

**Figure 3:**
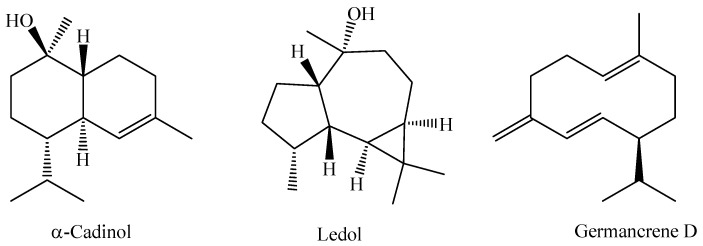
Major constituents of essential oil of *M. bella* leaves.

In a recent study, the chemical composition of the essential oil obtained from the leaves of an *M. bella* specimen collected during the rainy season in the Assis State Forest, São Paulo State, Brazil, was analyzed. The research indicated that the essential oil of *M. bella* is mainly composed of oxygenated sesquiterpenes α-cadinol (14.4%), ledol (12.2%), and the sesquiterpene hydrocarbon germacrene D (11.8%) [92]. The chemical structures of α-Cadinol, Ledol, and Germancrene D from *M. bella* are shown in Figure 3.

The species *M. laruotteana* Camb. is commonly known as “cambuí” in the southern and southeastern regions of Brazil. In the Amazon, it is also recognized by popular names such as “murta” and “smooth-skinned guava”. The species is a shrub or small tree that not only occurs in the southern, southeastern, and central-western regions of Brazil but also has a wide distribution in the states of Pará, Tocantins, Maranhão, Mato Grosso, Goiás, Distrito Federal, Mato Grosso do Sul, Minas Gerais, Espírito Santo, São Paulo, Paraná, and Santa Catarina [18].

**Figure 4:**
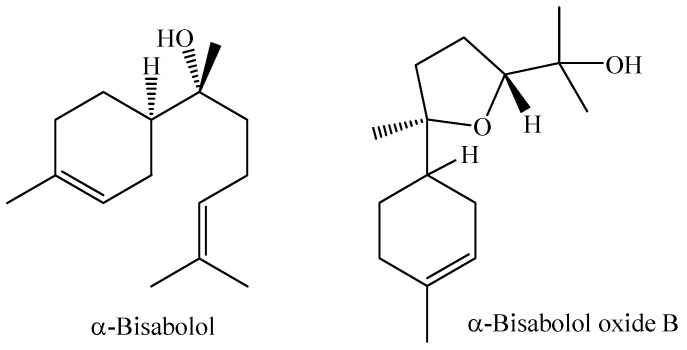
Main chemical constituents of the essential oil from *M. laruotteana* fruits.

The flowers of *M. laruotteana* are white and strongly aromatic, while the green fruits contain essential oil, which decreases as they ripen, giving rise to small edible fruits with a purple color. This species occurs in upland areas and floodplain forests, and morphological variations associated with different environments where individuals occur are observed. The study of the essential oil obtained by hydrodistillation from the green fruits of the species *M. laruotteana* Camb. showed that the major constituents are α-bisabolol (23.6%) and α-bisabolol oxide B (11.5%) [93]. Chemical structures of the compounds α-Bisabolol and α-Bisabolol oxide B are shown in Figure 4.

α-Bisabolol is the main constituent of the *M. laruotteana* essential oil and is also reported to have hypoglycemic properties. A study reports that α-bisabolol has antidiabetic properties related to reducing insulin resistance, glucose intolerance, plasma triacylglycerol, and LDL/VLDL cholesterol. Other factors such as the modulation of molecular pathways involved in the expression of target genes such as PPARs (Peroxisome proliferator-activated receptors) are among the properties described for this molecule [94].

**Figure 5:**
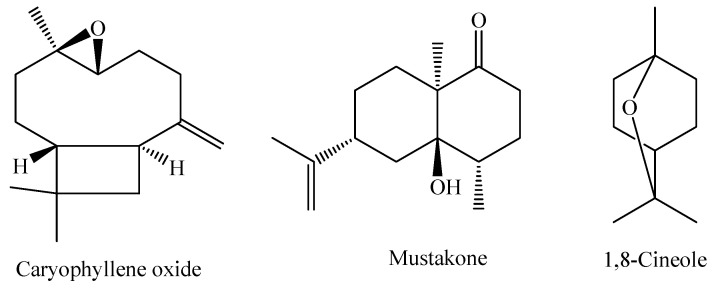
Major constituents of the essential oil of *M. pubiflora*.

The species *M. pubiflora* DC. is widely found in South America, especially in the region ranging from Brazil to Paraguay. It is a shrub that predominantly occurs in tropical and humid biomes [86]. The essential oil obtained from the fresh leaves of *M. pubiflora* shows the oxygenated sesquiterpene caryophyllene oxide as the major component (22.2%), with other components present in smaller amounts, such as mustakone (11.3%), 1,8-cineole (5.4%), and tryciclene (5.3%) [95]. The chemical structures of the main constituents of the essential oil from *M. pubiflora* leaves are shown in Figure 5.

The main constituent of the *M. publiflora* essential oil is caryophyllene oxide (caryophyllene-type structure). The antihyperglycemic activity of compounds with a caryophyllene-type structure has been reported in the literature. One study evaluated the activity of the compound β-caryophyllene in streptozotocin (STZ)-induced diabetic rats. The results showed that administration of β-caryophyllene at a dose of 200 mg/kg (body weight) had significant antidiabetic effects, demonstrating beneficial effects on glucose homeostasis in diabetic rats. Therefore, the hypoglycemic activity may be associated with the presence of a compound with a similar structure to this one but not only this one [89].

**Figure 6:**
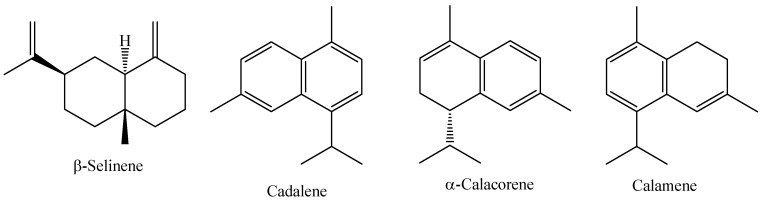
Major constituents of the essential oil of *M. sylvatica* fresh leaves.

*Myrcia sylvatica* (G. Mey) DC. is a species widely distributed in South America, from the Guianas to Brazil. This species is popularly known in the Amazon region as “murta”, “kumate-folha-miúda”, or “murtinha”, and it is also known as the “insulin plant” because local communities empirically use infusions of various parts of these plants to treat diabetes [82]. Res Research carried out with *Myrcia loranthifolia* grown in Atlantic Forest and Dry Forest forests in Brazil, showed that the essential oil of this species has an average yield of 0.23 to 0.33%, with the major compounds identified being (*E*)–caryophyllene (47.80%), Cis-calamenene (11.40%), and germacrene D (10.07%) [96]. The chemical structure of the main constituents of the essential oil of fresh *M. sylvatica* leaves are shown in Figure 6.

Another species known for its traditional use as a hypoglycemic agent is *Mycia*. It is a tree species that occurs in the Amazon biome in the northern regions, especially in the states of Amazon [9]. In the central-western region, it is commonly found in the state of Mato Grosso, in both upland forest and floodplain areas. This species is considered a medicinal plant in the Amazon region, and infusions of its leaves are used to prepare teas that are beneficial for consumption during pregnancy and for treating diabetes [9]. 

**Figure 7:**
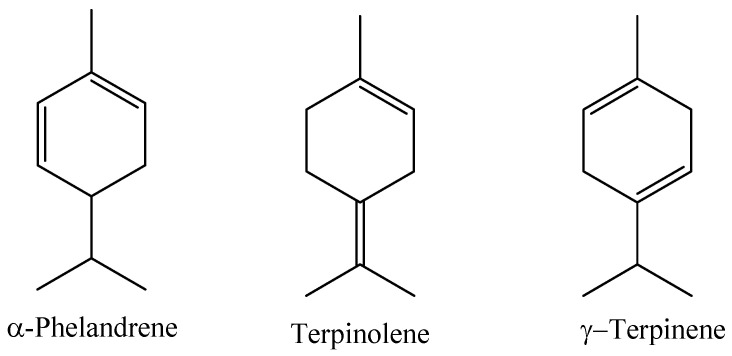
Major constituents of the essential oil of *M. paivae* leaves.

A study evaluating the chemical composition of the essential oil obtained by hydrodistillation of the leaves of a *Myrcia paivae* specimen collected in the municipality of Peixe-boi, Pará State, Brazil, identified the main constituents as compounds of the class of monoterpene hydrocarbons (77.93%), followed by sesquiterpene hydrocarbons (10.16%) and oxygenated monoterpenes (7.97%). In this study, the compounds α-phellandrene (14.69%), terpinolene (14.70%), and γ-terpinene (9.64%) were identified as the major ones [7]; the majority compounds can be seen in Figure 7.

In a study investigating the effect of α-Phelandrene, a major constituent in the essential oil of *M. paivae*, on the function of adipocytes under conditions of insulin resistance, the reduction in glucose uptake by insulin-resistant 3T3-L1 adipocyte cells was evaluated. The 3T3-L1 adipocytes were exposed to 25 µM glucose and insulin (0.6 nM) for 24 h. The author reported that α-Phelandrene contributed to benefiting the functions of adipocytes under conditions of insulin resistance. Cells treated with alpha-Phelandrene (65 µM) for 24 h showed a significant improvement in glucose uptake in the adipocytes of 3T3-L1 cells, with values of 11.31 mm/L, a result similar to that presented by the group treated with rosiglitazone [97]. Terpinolene, which is the second main constituent of the *Myrcia paivae* essential oil, is a substance whose activity in reducing blood glucose levels (mg/dL) has been described in the literature. The author reported that an extremely significant reduction in blood glucose levels was observed in diabetic female rats treated with 12.5 and 25.0 mg/kg of the terpene terpinolene for 4 weeks. In the group treated with 12.5 mg/kg of the substance, the animals had a blood sugar level of 580 mg/dL, and after treatment, the value was reduced to approximately 290 mg/dL. A similar result was observed for the group treated with 25 mg/kg of the terpene; the value before treatment was approximately 500 mg/dL and after treatment, 200 mg/dL [98]. In in vitro research involving various types of terpenes commonly found in essential oils, such as myrcene, eugenol, eucalyptol, β-pinene, citrol and terpineol, the effect and potential benefits in the prevention and treatment of diabetes were evaluated based on the action of these compounds against the α-amylase enzyme (enzyme responsible for hydrolyzing oligosaccharides into monosaccharides, increasing blood sugar levels). In this study, solutions were prepared containing different concentrations (µM.cm^−3^) of the selected terpenes. The results indicated that terpenes, which are the primary components of essential oils, have the potential to inhibit α-amylase to a significant degree, with the most efficient terpene concentrations ranging from 1.16 μmol.cm^−3^ for β-pinene to 5.50 μmol.cm^−3^ for eugenol [99].

The species mentioned above are particularly noteworthy for their popular uses by traditional indigenous communities and for their hypoglycemic activity. Studies highlighting the chemical composition of the leaves, flowers, and fruits, as well as the essential oils of these species, provide the scientific community with knowledge about this natural wealth. Most of these *Myrcia* species, as the text highlights, occur in Brazilian biomes, being predominantly distributed in the Cerrado, Caatinga, and the Amazon, and constitute a natural wealth that is still little known.

The data cited in the previous text reveal interesting activities for the essential oils obtained from *Myrcia* species as natural pharmacological agents, which could be important for the development of phyto-formulations against diseases such as DM2. The essential oils from the leaves of these *Myrcia* species, popularly used as hypoglycemic agents, can be considered a potential source of pharmacological agents, which could be used in formulations for the treatment of diabetes, Alzheimer’s, and diseases linked to oxidative stress and applied as active ingredients in phyto-formulations as an alternative to the use of synthetic drugs.

## 7. Conclusions

The *Myrcia* genus is a promising source of essential oils, whose chemical composition is characterized by great complexity, which depends on climate location and seasonal factors, among others. The results obtained by the consulted studies indicate that *Myrcia* essential oils can constitute a promising source of bioactive compounds such as monoterpenes and sesquiterpenes with different biological activities including antioxidant, antimicrobial, phytotoxic, insecticidal, and hypoglycemic. All these effects can attract the attention of several industrial sectors, such as the pharmaceutical and cosmetic industries, which open up a vast field of therapeutic possibilities. The *M. eximia* sample A shows a high quantity in percentage of hexanal (26.09%) and (*E*)-caryophyllene (20.3%), and sample B, caryophyllene oxide (22.16%) and α-copaene. Furthermore, *M. hatschbachii* exhibits trans-calamenene (19.10%) and (*E*)-caryophyllene (10.96%), whilst *M guianensis* is notable for its methyl salicylate (11.13%) and Geraniol (8.03%). Among the variety of *Myrcia* species, (*E*)-caryophyllene and caryophyllene oxide are substances most commonly found in the various *Myrcia* species. These complex chemical compositions are present in several *Myrcia* plant species and sections, demonstrating the genetics and environmental complexity of plants, as well as the impact of geographical and climatic variables. Understanding these intricacies is vital for pragmatic implementations in the fields of medicine, cosmetics, and other domains. Promoting the sustainable use of these vital resources all depends on protecting these species and their habitats. 

## Figures and Tables

**Table 2:** Antioxidant activity of *Myrcia* genus.

*Myrcia* Species	Major Components of EO	Method	(%) Inhibition	References
*M. tomentosa*	γ-elemene (12.52%), germacrene D (11.45%), and (*E*)-caryophyllene (10.22%)	Trolox equivalent antioxidant capacity (TEAC) by 1,1-diphenyl-2-picrylhydrazyl (DPPH)	0.333 ± 0.247% (ABTS) and 208.5 ± 0.940% (DPPH)	[3]
*M. hatschbachii* D. Legrand	*trans*-calamenene (19.10%), (*E*)-caryophyllene (10.96%), and spathulenol (5.03%)	DPPH scavenging method; phosphomolybdenum complex formation assay	9.14 ± 0.33% (DPPH) (DPPH)	[42]
*M. floribunda*	α-phellandrene (22.19%), 1,8-cineole (23.30%), terpinolene (22.23%), o-cymene (7.04%), and γ-terpinene (5.87%)	2,2′-azino-bis (3-ethylbenzothiazoline-6-sulfonic acid) diammonium salt (ABTS) and DPPH Assay	53.27 ± 8.27% (ABTS) and 81.91 ± 3.46% (DPPH)	[10]
*M. sylvatica*	(*Z*)-α-*trans*-bergamotene (24.57%), α-sinensal (13.44%), (*Z*)-α-bisabolene (8.33%), α-*trans*-bergamotene (7.06%), and β-*trans*-bergamotene (5.07%)	ABTS and DPPH assay	7.20 ± 0.72% (ABTS) and 80.55 ± 2.00% (DPPH)	[10]
*M. paivae* O.Berg	terpinolene (14.70%), α-phellandrene (14.69%), γ-terpinene (9.64%), sylvestrene (7.62%), α-thujene (6.46%), α-pinene (6.39%)	(ABTS) and (DPPH) assay	0.886 ± 0.226% (ABTS) and 2.90 ± 0.083% (DPPH)	[7]
*M. oblongata*	not analyzed	free radical reduction method using DPPH radical	90.18	[55]
*M. oblongata*	α-pinene (36.81%), β-myrcene (13.99%), silvestrene (11.28%), and β-pinene (9.74%),	DPPH (2,2-diphenyl-1-picrylhydrazyl) and ABTS (2,2′-azino-di-(3-ethylbenzthiazoline sulfonic acid)) radical scavenging methods and antioxidant power were assessed by Ferric reducing antioxidant power (FRAP)	88.08 ± 1.3% (ABTS) and 55.74 ± 2.4% (DPPH)	[56]
*M. splendens*	*trans*-nerolidol (67.81%), α-bisabolol (17.51%), and β-caryophyllene (4.21%)	DPPH-HPTLC (1,1-diphenyl-2-picrylhydrazil—high performance thin layer chromatography) bioautographic assay and Spectrophotometric DPPH assay	43,537.00 ± 15% (DPPH)	[48]
*M. splendens*	myrcene (7.9%), α-copaene (4.5%), *E*-caryophyllene (45.8%), α-humulene (5.0%), and germacrene B (5.9%)	DPPH Radical Scavenging Assay	28.4 ± 7.1 (DPPH)	[57]
*M. sylvatica*	*E*-caryophyllene (10.0%), γ-elemene (12.5%), bicyclogermacrene (5.0%), and germacrene B (24.5%)	DPPH Radical Scavenging Assay	18.5 ± 3.5 (DPPH)	[57]

**Table 3:** Synthesis of antifungal and antibacterial activities and the main constituents of essential oils from *Myrcia* species.

Species	Main Constituents (%Area)	Activity Against	Reference
Nine plants of *M. ovata* Cambessedes	Geraniol (74.37 to 0.45%) Nerolic acid (73.97 to 6.33%) Geranial (40.10 to 0.10%) 1,8-Cineole (33.03 to 0.75%) Neral (28.39 to 0.11%) Isopulegol (27.50 to 2.30%) (*E*)-Nerolidol (20.24 to 0.29%) Linalool (19.61 to 0.53%) Citronellal (0 to 9.19%) Iso-isopulegol (0 to 10.29%)	Results of diameters of inhibition zone in mm Gram-positive bacteria *Staphylococcus aureus* (11–28.5) *Bacillus cereus* (10–25.5) *Bacillus subtilis* (13.5–30) *Enterococcus faecalis* (7.5–22) Gram-negative bacteria *Pseudomonas aeruginosa* (8–30) *Serratia marcensces* (6–27) *Escherichia coli* (8–24) *Salmonella enteritidis* (6–26.5)	[40]
* M. hatschbachii * D. Legrand	Trans-calamanene (19.10) (*E*)-Caryophyllene (10.96) Spathulenol (5.03)	Results of MIC in µg/mL Gram-positive bacteria *Enterococcus faecalis* (500) *Staphylococcus aureus* (1000)	[42]
* M. multiflora *	(A) α-Bulnesene (26.79%) Pogostol (21.27%) δ-Amorphene (6.76%) (B) (*E*)-Nerolidol (44.4%) (*E*)-γ-Bisabolene (10.64%) (*E*,*E*)-α-Farnesene (8.19%) (C) (E)-Nerolidol (92.21%)	Results of diameters of inhibition zone in mm Leveduras (A–B–C) *Candida albicans* (8–9–7) *C. tropicalis* (8–11–8) *C. famata* (6–10–8) *C. krusei* (9–10–8) *C. auris* (9–10–8)	[4]
*M. oblongata* DC	Uninformed	Results of MIC/MBC in µg/mL Gram-negative bacteria *Salmonella Albany* (3500/7000) *S. braenderup* (3500/7000) *S. gafsa* (1750/3500) *S. heidelberg* (3500/7000) *S. idikan* (1750/3500) *S. Lexington* (437.5/3500) *S. livingstone* (3500/3500) *S. montevideo* (3500/-) *S. saintpaul* (875/1750) *S. senftenberg* (3500/7000)	[55]
*M. ovata*	Geranial (50.4%) Neral (35.8%) 1,8-Cineole (4.8%) α-Terpineol (1.1%)	Results of diameter of inhibition zone in mm/MIC (%)/MBC (%) Gram-positive bacteria *Enterococcus faecalis* (36/0.031/0.031) *Staphylococcus aureus* (23/0.25/−0.5) *Streptococcus pneumoniae* (25/0.0625/0.25) Gram-negative bacteria *Escherichia coli* (21/1/1) *Helicobacter pylori* (0/0/0) *Pseudomonas aeruginosa* (16/>1/>1) *Salmonella choleraesuis* (26/0.5/0.5) *Salmonella choleraesuis* (26/0.5/0.5) Yeast *Candida parapsilosis* (30/0.031/-)	[60]
* M. splendens * (Sw.) DC. (sin. *M. fallax* (Rich.) DC.)	Trans-nerolidol (67.81%) α-Bisabolol (17.51%)	Results of MIC in µg/mL Gram-positive bacteria *Clavibacter michiganensis* subsp. *nebraskensis* (125) *Enterococcus faecalis* (2000) *Listeria grayi* (1000) *Staphylococcus aureus* (1000) *Staphylococcus epidermidis* (1000) Gram-negative bacteria *Agrobacterium tumefaciens* (500) *Agrobacterium vitis* (2000) *Pseudomonas syringae* pv. *syringae* (250) *Escherichia coli* (>2000) *Pseudomonas aeruginosa* (>2000)	[48]

MIC = Minimum Inhibitory Concentration; MBC = Minimum Bactericidal Concentration.

## Data Availability

Data are contained within the article.
